# Initiation of peritoneal dialysis in a patient with chronic renal failure associated with tetralogy of Fallot: a case report

**DOI:** 10.1186/s12882-020-01939-x

**Published:** 2020-07-15

**Authors:** Tetsuya Abe, Togo Aoyama, Keiko Sano, Ryoma Miyasaka, Takuya Yamazaki, Yukari Honma, Hiroshi Tominaga, Maoko Ida, Aya Arao, Mayuko Sakakibara, Keiko Hashimoto, Haruka Takahashi, Takeshi Sakai, Shokichi Naito, Toshimi Koitabashi, Takashi Sano, Yasuo Takeuchi

**Affiliations:** 1 Department of Nephrology, Kitasato University School of Medicine, 1-15-1 Kitasato, Minami, Sagamihara, Kanagawa 252-0375 Japan; 2 Department of Cardiovascular Medicine, Kitasato University School of Medicine, 1-15-1 Kitasato, Minami, Sagamihara, Kanagawa 252-0375 Japan

**Keywords:** Peritoneal dialysis, Tetralogy of Fallot, Chronic renal failure

## Abstract

**Background:**

Tetralogy of Fallot is the most common cyanotic congenital heart disease. Patients with the condition have a high risk of developing chronic kidney disease. Treatment of kidney disease in patients with complex hemodynamics presents unique challenges. However, there are very few reports on the treatment of end-stage renal failure in patients with tetralogy of Fallot.

**Case presentation:**

We present a rare case of peritoneal dialysis in a 47-year-old man with tetralogy of Fallot who had not undergone intracardiac repair. Peritoneal dialysis successfully removed fluids and solutes without adversely affecting the patient’s hemodynamics. Our patient was managed with peritoneal dialysis for 5 years before he succumbed to sepsis secondary to digestive tract perforation.

**Conclusions:**

In this paper, we discuss the importance of monitoring acid–base balance, changes in cyanosis, and hyperviscosity syndrome during peritoneal dialysis in patients with tetralogy of Fallot. Lower leg edema and B-type natriuretic peptide level were useful monitoring parameters in this case. This case illustrates that with attention to the patient’s unique requirements, peritoneal dialysis can provide successful renal replacement therapy without compromising hemodynamics in patients with tetralogy of Fallot.

## Background

Tetralogy of Fallot is a cyanotic heart disease characterized by a ventricular septal defect, right ventricular hypertrophy, pulmonary artery stenosis, and overriding aorta. Chronic renal dysfunction is a late complication that occurs after treatment of tetralogy of Fallot [1]. Diabetes, hypertension, and use of diuretics are risk factors for the development of renal dysfunction. There are no reports of renal dysfunction in a patient with cyanotic tetralogy of Fallot who did not undergo intracardiac repair. Details of this condition are unknown.

There are few reports of patients with tetralogy of Fallot and end-stage renal failure. We report a rare case of peritoneal dialysis (PD) in a patient with chronic renal failure resulting from tetralogy of Fallot.

## Case presentation

Our patient was a 47-year-old man who was diagnosed with tetralogy of Fallot 3 months after birth. He had a Waterston operation at 9 months of age and a Blalock–Taussig procedure at 1 year, 10 months of age. At age 10, the patient underwent surgery to ligate the ascending aorta–right pulmonary artery shunt path. He did not undergo intracardiac repair. The main pulmonary artery was completely occluded from the right ventricular outflow tract. The right-to-left shunted blood ejected from the left ventricle through the ventricular septal defect and merged with oxygenated blood via the shunt path from the left subclavian artery to the left pulmonary artery.

Renal function decreased when the patient was 38 years of age. Home oxygen therapy at night was initiated 1 year before the patient presented at our hospital; a β-blocker (carvedilol, 2.5 mg/day) was started 6 months before presentation. The patient did not experience breathing difficulty in his daily life (New York Heart Association Class II) and was able to work. A PD catheter was inserted in April 2015. Renal function gradually decreased and exacerbation of lower leg edema was observed, so PD was started. The patient’s height was 158 cm, his weight was 54 kg, his body temperature was 36.3 °C, his blood pressure was 136/69 mmHg, his pulse rate was 81 beats/min, and his oxygen saturation was 81% in room air.

Laboratory testing was performed on admission. Urinalysis dipstick examination indicated 4+ protein (8 g/g Cr) and was negative for blood. Arterial blood gas analysis showed a pH of 7.278, PaCO_2_ of 43 mmHg, PaO_2_ of 52 mmHg, bicarbonate of 19.7 mmol/L, and base excess of − 6.9 mmol/L. Chest X-ray showed a cardiothoracic ratio of 63%, indicating cardiac enlargement. Abdominal ultrasonography showed mild atrophy of the bilateral kidneys, increased cortical brightness, and cysts. Left ventricular findings of cardiac ultrasonography included a left ventricular end-diastolic diameter of 44 mm, left ventricular end-systolic diameter of 28 mm, left ventricular ejection fraction of 71%, septal thickness of 26 mm, and rear wall thickness of 11 mm. The valve findings were mild aortic regurgitation, trivial mitral regurgitation, moderate tricuspid regurgitation, and pulmonary artery closure. The main pulmonary artery was not visible from the right ventricular outflow tract. The ventricular septal defect was 19–22 mm in diameter; there was almost right-to-left shunting across the defect, with only a slight left-to-right shunt. After Waterston operation there was good flow through the shunt from the ascending aorta to the right pulmonary artery. After left Blalock–Taussig shunt operation there was decreased flow velocity from the left subclavian artery to the left pulmonary artery.

**Fig. 1:**
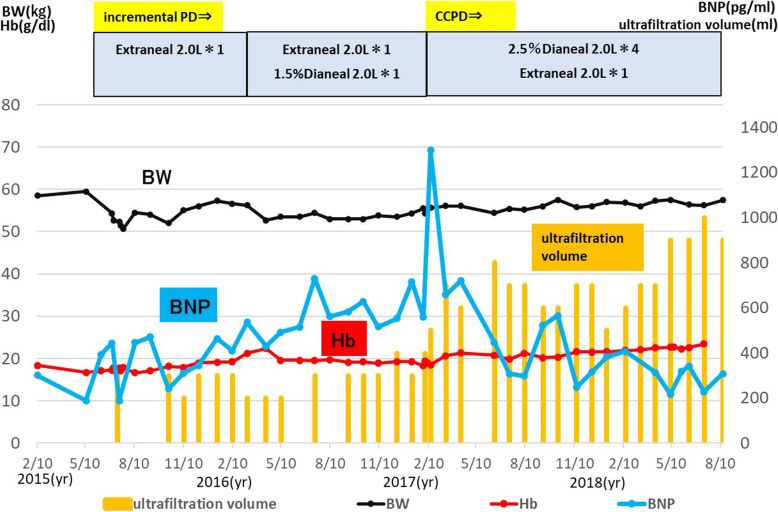
Changes in peritoneal dialysis protocol and clinical parameters. At PD initiation, the patient was treated with incremental PD only at night and urine volume was maintained at 600–1000 ml/day. Peritoneal dialysate was subsequently added during the day because of lack of solutes and poor ultrafiltration volume. In 2017, the patient had weight gain and leg edema, his BNP increased to 1300 pg/ml, and his fast PET test result was high average. After the switch to CCPD, body fluid management improved. BW, body weight; PD, peritoneal dialysis; CCPD, continuous cyclic peritoneal dialysis; BNP, brain natriuretic peptide

In July 2015, PD was started. Serum creatinine (Cr) at the start of PD was 5.5 mg/dl. The fast peritoneal equilibrium test (PET) result was low average. From November 2015 (4 months after the start of PD), B-type natriuretic peptide (BNP) gradually increased, water removal was 200–300 mL/day, and body weight increased. In March 2016 (8 months after starting PD), the PD fluid protocol was changed. In February 2017, the patient developed lower leg edema and his BNP increased to 1300 pg/mL, so treatment was changed to continuous cycling PD. With increased water removal, the edema resolved and BNP rapidly decreased. Changes associated with cyanosis included a hemoglobin level of 18–20 g/dL, indicating polycythemia; the patient also experienced headache. Phlebotomy treatment was performed four times. Because of concerns about iron deficiency resulting from phlebotomy, hemostasis therapy was discontinued, after which the patient’s headache improved (Fig. 1).

**Fig. 2:**
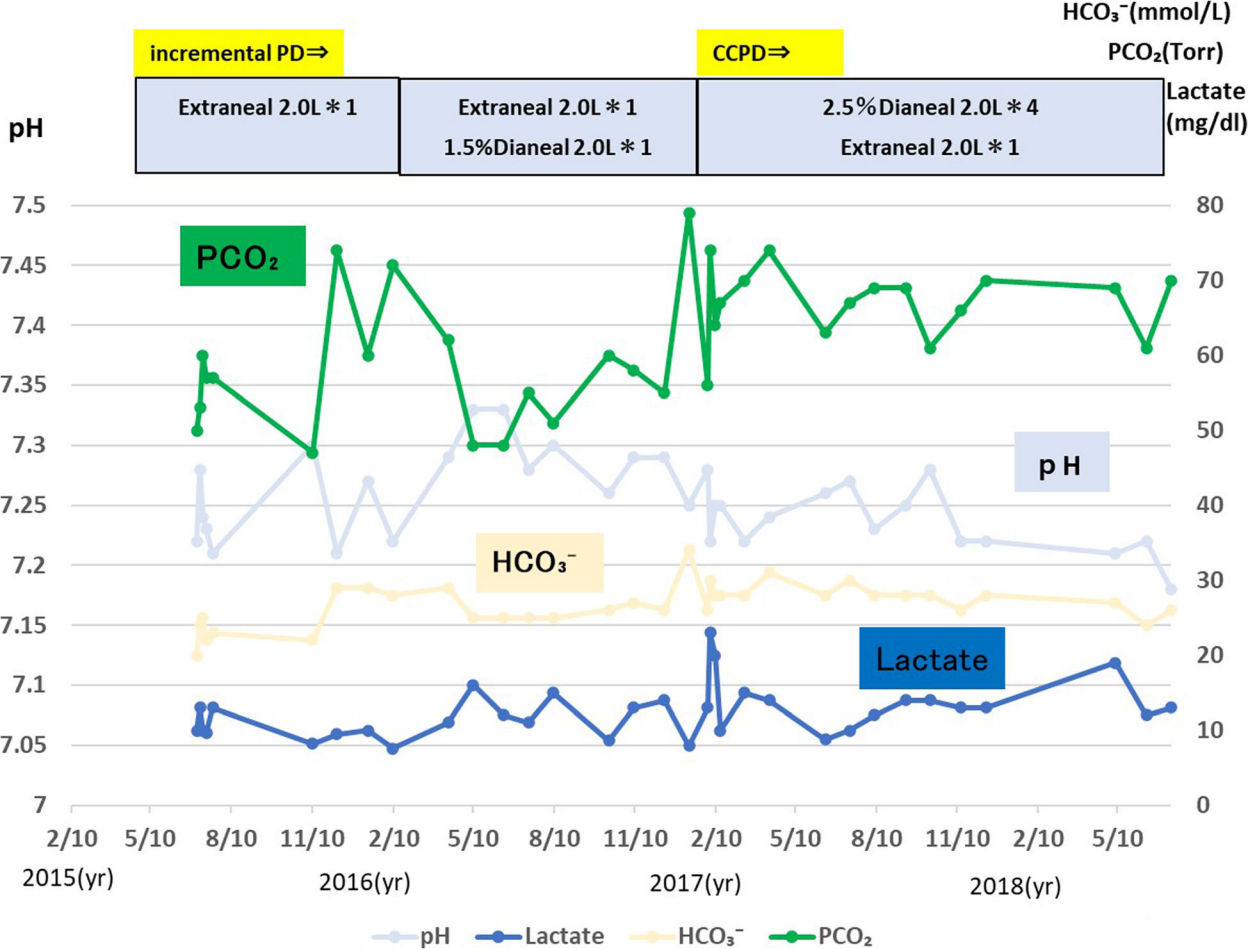
Changes in venous blood gases after initiation of peritoneal dialysis. The serum bicarbonate level at the start of PD in July 2015 was 20 mmol/L. This value increased to 25–30 mmol/L with the change in PD fluid protocol. Venous blood PCO_2_ increased with the start of PD. The lactic acid level remained almost unchanged during the course of treatment. PD, peritoneal dialysis; CCPD, continuous cyclic peritoneal dialysis

The serum bicarbonate level at the start of PD in July 2015 was 20 mmol/L. This value increased to 25–30 mmol/L with the change in PD fluid protocol, and venous blood PCO_2_ increased with the start of PD (Fig. 2).

The target SpO_2_ was adjusted to 70–85%; SpO_2_ was unchanged after PD. SpO_2_ decreased with the complication of heart failure, but improved with fluid management. Periodic echocardiography showed that PD initiation had little effect on hemodynamics.

There were no PD-related complications.

In July 2019, 5 years after the start of dialysis, the patient was admitted to the hospital for treatment of abdominal pain and dyspnea. Abdominal CT revealed a diverticulum of the colon in the hepatic curvature of the transverse colon. Surgery was considered to carry a high risk of respiratory and hemodynamic breakdown in this patient. On the basis of abdominal findings and CT findings, it was concluded that conservative treatment with antibiotics and fasting was appropriate. With the consent of the family, laparotomy was not performed and conservative treatment was initiated. *Streptococcus salivarius* was subsequently detected in culture of the PD effluent and MRSA was detected in blood culture. Antibiotic treatment based on sensitivity testing was started and the peritoneal catheter was removed, but there was no improvement. The patient developed septic shock and died 27 days after admission.

## Discussion and conclusions

We experienced a rare case of PD in a patient with end-stage renal failure resulting from cyanotic tetralogy of Fallot without intracardiac repair. In this patient with complex hemodynamics, PD had minimal effect on the abnormal circulatory dynamics and effectively removed water and solutes. However, problems such as changes in acid–base balance associated with PD initiation, effects on cyanosis, and hyperviscosity syndrome associated with secondary polycythemia did occur.

Renal replacement therapy in patients with tetralogy of Fallot requires careful selection because of hemodynamic problems. There have been three prior reported cases of renal replacement therapy in patients with tetralogy of Fallot. The first case was a 44-year-old man with serum Cr of 2.4 mg/dL who was treated with hemodialysis because of fluid retention [2]. The second case was a 33-year-old woman with serum Cr of 8.0 mg/dL and uremia who was initially treated with hemodialysis with a temporary indwelling catheter and was later switched to PD [3]. The third case was acute renal tubular necrosis resulting from hypotension-induced renal damage; hemodialysis was introduced, but the patient died soon after initiation of treatment [4]. To date, there is no clear evidence on the best blood purification method for renal replacement therapy in patients with tetralogy of Fallot.

The problem with hemodialysis in patients with tetralogy of Fallot is that temporary or permanent catheterization is likely to cause complications such as pulmonary embolism, sepsis, infective endocarditis, and paradoxical embolism [5–8]. In addition, in patients with tetralogy of Fallot with complex hemodynamics, the creation of peripheral vascular access may cause heart failure [9].

Problems with PD include the need to insert a catheter under general anesthesia. Patients with tetralogy of Fallot have many collateral blood vessels resulting from advanced cyanosis. Bleeding from these collateral vessels is expected when the incision is made and it is highly likely that blood transfusion will be necessary. In addition, when PD is started, the diaphragm elevation resulting from infusion of PD solution may exacerbate respiratory failure.

In this case, we considered the effects of hemodialysis on cardiac function and expected it would have a high likelihood of complications and impacts on hemodynamics. Therefore, PD was selected.

Systemic abnormalities in cyanotic congenital heart disease include polycythemia, hyperviscosity syndrome, coagulopathy, cyanosis nephropathy, cerebrovascular disorders, pulmonary hemorrhage, hyperuricemia, gout attacks, gallstones, clubbed fingers, and infective endocarditis [10]. Management of systemic complications is essential in clinical practice for adults with congenital heart disease.

In addition to the possible complications of surgical intervention in this population, injection of PD fluid may increase abdominal pressure and exacerbate respiratory acidosis. The progression of acidosis can worsen cyanosis by shifting the oxygen dissociation curve to the right. Dianeal®, a lactic acid-buffered neutral dialysis solution, was used as the PD solution in this case. Because Dianeal® has a high lactic acid content of 40 mEq/L as a buffer, there is an increased risk of peritoneal deterioration and a concern that metabolic acidosis could be excessively corrected.

When managing complications of renal failure, it is necessary to recognize and understand the management of anemia. In patients with cyanotic congenital heart disease, erythropoietin secretion increases as a result of decreased oxygen saturation and oxygen-carrying capacity in tissues, resulting in secondary red blood cell increase. A hemoglobin level of 14 g/dL in these patients indicates iron deficiency anemia. It is reported that a patient with oxygen saturation of 85% requires a hemoglobin level of 20 g/dL or higher. If iron deficiency is present (mean erythrocyte volume < 80 fL), iron should be administered [11, 12]. In this patient, iron was not used and management was performed without using erythropoietin-stimulating agents.

We experienced a rare case of PD in a patient with chronic renal failure in the context of tetralogy of Fallot, which is associated with complex hemodynamics. PD can have minimal effects on hemodynamics in patients with tetralogy of Fallot. Useful monitoring parameters were lower leg edema and BNP. Attention should be paid to the effects of PD on acid–base balance.

## Data Availability

Not applicable.

## Abbreviations

PD: Peritoneal dialysis
Cr: Creatinine
BNP: Brain natriuretic peptide
BW: Body weight
